# Kakkonto, shosaikoto, *Platycodon grandiflorum* root, and gypsum (a Japanese original combination drug known as saikatsugekito): Pharmacological review of its activity against viral infections and respiratory inflammatory conditions and a discussion of its applications to COVID‐19

**DOI:** 10.1002/tkm2.1258

**Published:** 2020-10-11

**Authors:** Ryutaro Arita, Rie Ono, Natsumi Saito, Shin Takayama, Takao Namiki, Takashi Ito, Tadashi Ishii

**Affiliations:** ^1^ Department of Kampo Medicine Tohoku University Hospital Sendai Japan; ^2^ Department of Education and Support for Regional Medicine Tohoku University Hospital Sendai Japan; ^3^ Department of Kampo and Integrative Medicine Tohoku University Graduate School of Medicine Sendai Japan; ^4^ Department of Japanese‐Oriental (Kampo) Medicine Graduate School of Medicine, Chiba University Chiba Japan; ^5^ Akashi Clinic Tokyo Japan

**Keywords:** COVID‐19, kakkonto, Kampo medicine, saikatsugekito, shosaikoto, viral infection

## Abstract

**Aim:**

Traditional Japanese (Kampo) medicine has been used to treat viral infectious diseases. In particular, saikatsugekito (a combination drug of kakkonto, shosaikoto, *Platicodon glandiflorum* root, and gypsum) has been reported to be useful during the past influenza pandemic. The severe acute respiratory syndrome coronavirus 2 (SARS‐CoV‐2) has spread worldwide, causing the novel coronavirus disease (COVID‐19) to emerge as a pandemic. In this article, we conducted a literature review on the pharmacological activities of the components present in saikatsugekito against viral infection and respiratory inflammation.

**Methods:**

We searched PubMed and the Cochrane Library for English articles, as well as Ichushi and J‐stage for Japanese articles. Articles published until January 1, 2000 were retrieved using the keywords ‘kakkonto’, ‘shosaikoto’, ‘Platycodon’, and ‘gypsum’. We then extracted articles on basic research investigating viral infections, inflammation, cytokine, the immune response, and lung tissue damage.

**Results:**

We extracted 28 eligible articles. Kampo medicines have antiviral activities by interfering with the attachment, internalization, replication, progeny virion release, and cell‐to‐cell spreading of single‐strand RNA viruses. They also enhance the immunomodulating activities of the host, including cytokine production, regulation of multiple immune cells, and protection from lung tissue injury. Furthermore, Kampo medicine has been found to regulate body temperature and airway mucin release.

**Conclusion:**

The results demonstrated that Kampo medicine has therapeutic activities against single‐strand RNA virus infections and respiratory inflammation, and may also have activities against SARS‐CoV‐2. Further research is required to investigate the activity of Kampo medicines, such as saikatsugekito, against SARS‐CoV‐2.

## BACKGROUND

Japanese traditional (Kampo) medicine has been used to treat infectious diseases for a long time. The classic text Shokanron (Shanghanlun in Chinese) is one of the most important textbooks for infectious diseases in traditional Chinese and Kampo medicine. It contains many formulas for infectious diseases used for the past 1800 years and even in recent times. For example, during the last worldwide influenza pandemic in 1918 (the so‐called Spanish flu), many patients were treated with Kampo formulas, such as kakkonto (KKT), shosaikoto (SST), daiseiryuto, shoseiryuto, kososan, shomakakkonto, or saikatsugekito (SGT) according to the symptoms or clinical stage of the patients [1, 2]. Many of them are recorded in Shokanron.

Among Kampo medicines, SGT in particular has been used to treat severe infectious diseases in Japan since Sohaku Asada reconstructed it in the 19th century based on the original Shokan un'yo (Shanghan yunyao in Chinese) [3]. It has been reported that none of the patients who were administered SGT for the Spanish flu died [1]. This formula is indicated for conditions with severe symptoms, such as headache, chill, fever, malaise, arthralgia, thirst, dry nose, nausea, appetite loss, and even dysphoria, which are commonly observed in the acute phase of influenza‐like illness. From the perspective of traditional medicine's ‘six‐stage pattern theory’ [4, 5], such a condition is considered an overlap or combination of ‘early yang stage ^(^™^1)^’, ‘middle yang stage ^(^™^1)^’, and even ‘late yang stage ^(^™^1)^’ patterns, which suggests a complicated and severe inflammatory state of the acute phase [6, 7, 8].

A novel coronavirus disease (COVID‐19) outbreak has emerged in Wuhan, China since December 2019 [9], and the World Health Organization declared the rapidly spreading outbreak a pandemic on 11 March 2020 which is currently threatening global health. In Japan, over 13 000 confirmed infected cases and over 400 deaths have been reported as of 1 May 2020. Major clinical symptoms among COVID‐19 patients are fever, cough, and fatigue, while various minor symptoms include myalgia, sputum production, shortness of breath, chest pain, headache, sore throat, dizziness, anorexia, diarrhea, nausea, and vomiting [10, 11]. Some cases develop rapid pneumonia and fatal respiratory failure. In China, 81% of confirmed cases were classified as mild, 14% as severe, and 5% as critical, and the overall case fatality rate was 2.3% [12]. Severe disease leads to acute respiratory distress syndrome (ARDS), systemic inflammatory response syndrome, and multiple organ failure, which is induced by the cytokine release syndrome (CRS, so‐called cytokine storm). The pathophysiology of CRS is characterized by dysregulation of the immune response to pathogens, leading to cytokine overexpression [13]. Many potential therapeutic agents, including antiviral and antimalarial agents, protease inhibitors, and inhaled glucocorticoids have been administered in clinical trials [14, 15, 16, 17, 18], but their therapeutic efficacies are still unknown or controversial.

SGT used during the Spanish influenza is similar to a combination of KKT, SST, and gypsum (sekko) (Table 1). All the component crude drugs of SGT are listed in the Japanese Pharmacopeia 17th edition (JP) [19]. In a modern Japanese setting using Kampo extract formulas, SGT could be reproduced as a combination drug with KKT, SST, and kikyosekko. Kikyo is made of dried *Platycodon glandiflorum* roots (PR), and is usually used to treat sore throat, cough, phlegm, and other symptoms of respiratory inflammation. Sekko is usually used to treat various inflammatory conditions. Further drug information of each Kampo formula is listed in JP and STORK (http://mpdb.nibiohn.go.jp/stork/).

**Table 1:** Components crude drugs of kakkonto, shosaikoto, and saikatsugekito

Component crude drugs	*Pueraria* root	*Ephedra* herb	*Cinnamon* bark	Peony root	Jujube	Ginger	*Glycyrrhiza*	*Bupleurum* root	S*cutellaria* root	*Pinellia* root	Ginseng	Gypsum	*Platycodon* root	*Angelicadahurica* root	*Notopterygium*
**Saikatsugekito (Asada)**	✓	✓	✓	✓		✓	✓	✓	✓	✓	✓	✓			
Kakkonto	✓	✓	✓	✓	✓	✓	✓								
Shosaikoto					✓	✓	✓	✓	✓	✓	✓				
Shosaikotokakikyosekko					✓	✓	✓	✓	✓	✓	✓	✓	✓		
Saikatsugekito (Shokanrikusho)	✓		✓	✓		✓	✓	✓	✓	✓		✓	✓	✓	✓
Saikatsugekito (Shokanun'yo)	✓			✓	✓	✓	✓	✓	✓	✓	✓				

Saikatsugekito (Asada) is the Japanese original combination, and usually prescribed in Kampo clinical practice in Japan. In this review, we reproduce this combination using kakkonto and shosaikotokakikyosekko.

All the crude drugs are listed in the Japanese Pharmacopeia (17th edition).

It is important to evaluate if the original Japanese Kampo formula SGT, used during the Spanish influenza, can be used against COVID‐19 as well. No studies have been conducted on the pharmacological effects of SGT against viral infections and respiratory inflammatory conditions, such as Spanish flu or COVID‐19. Therefore, we tested our expectations of the effects of SGT by examining the pharmacological effects of KKT, SST, PR, and gypsum, which are the crude drugs present in SGT. This literature review provides the pharmacological activities of KKT, SST, PR, and gypsum on viral infections and inflammatory conditions, including their possible therapeutic activities against COVID‐19, and elucidates the life cycle of severe acute respiratory syndrome coronavirus 2 (SARS‐CoV‐2) and the immune response of the host.

## LITERATURE SEARCH

### Literature search methods

Using the keywords ‘kakkonto’, ‘shosaikoto’, ‘Platycodon’, or ‘gypsum,’ and their Chinese and Korean equivalents, we conducted a database search of PubMed and the Cochrane Library for articles written in English, while Ichushi and J‐Stage were used to find articles written in Japanese. The search was restricted to articles published since 1 January 2000.

### Selection criteria

Basic research on viral infections, inflammation, cytokines, the immune response, and lung tissue damage was selected from a pool of research articles published in English or Japanese. We excluded studies focusing on hepatitis virus infection, liver injury, or allergic immune responses, which would not be related to COVID‐19.

### Data extraction

Eligible articles were categorized by two independent researchers (RA and RO) who extracted and tabulated specific information from the articles.

## RESULTS

We extracted 28 eligible articles (KKT: 9; SST: 8; Platycodon: 11; gypsum: 2, respectively), but did not find reports on coronavirus. We reviewed the pharmacological activities of each drug as follows.

### Kakkonto (KKT)

KKT, known as Ge‐Gen‐Tang in Chinese and Galgeun‐Tang in Korean, is widely used to treat the common cold, fever, headache, neck stiffness, and diarrhea. In Japan, KKT has been used to treat viral infectious diseases, such as measles and influenza, for over hundreds of years. Kakkonto extract contains seven crude drugs: JP *Pueraria* root, JP *Ephedra* herb, JP Jujube, JP Cinnamon bark, JP Peony root, JP *Glycyrrhiza*, and JP Ginger [19]. KKT is often used in the super‐acute and acute phase of the common cold, which is called the ‘early yang stage pattern ^(^™^1)^’ in the traditional six‐stage pattern theory [4].

Chang *et al*. reported that the effectivity of KKT against human respiratory syncytial virus (HRSV) in upper and lower respiratory tract cell lines was time‐ and dose‐dependent. KKT inhibited viral attachment and internalization in these cells [20]. Inhibitory activity against HRSV attachment was observed when human host cells were treated with hot water extracts of fresh ginger, which is contained in ‘classical’ KKT, whereas dried ginger did not have the inhibitory activity [21]. Shirayama *et al*. reported that KKT, SST, and other Kampo formulas frequently used for the common cold had dose‐dependent inhibitory activity against the polymerase acidic protein endonuclease, which is essential for the acquisition of primers for viral mRNA transcription [22].

Muraoka *et al*. reported that the body temperature of KKT‐treated dogs increased 30 min after administration. The phagocytic activity of serum macrophages in dogs increased after administration of KKT. In the non‐infected condition, KKT could enhance the phagocytic activity of macrophages [23]. Kurokawa *et al*. reported that KKT administration reduced mortality and prolonged the survival of mice infected with H1N1 influenza. Viral yield in bronchoalveolar lavage fluid (BALF) decreased in the early phase in KKT‐administered mice. The levels of IL‐12 in BALF were increased by KKT administration in the early phase of infection. However, serum IL‐12 did not show a significant change [24]. IL‐12 promotes the differentiation of naïve T cells into Th1 cells, suggesting that KKT might develop a Th1 immune response and increase IL‐12 production only in infected organs, but might not enhance systemic inflammation in the early phase of infection. Geng *et al*. reported that KKT had greater inhibitory activity against H1N1 influenza infection in canine kidney (MDCK) cells when it was administered before infection compared with after infection. KKT targeted the viral attachment and replication stage rather than the internalization stage. Virus‐infected mice experiments revealed that the virus titers and the expressions of viral nucleoprotein in lung tissue were reduced in the KKT‐administered mice. KKT treatment improved histological changes in the lungs, such as narrowed alveolar space, thickened alveolar wall, lung congestion and infiltration of inflammatory cells. KKT reduced the expression of IL‐1α, IL‐6, and TNF‐α, which are pro‐inflammatory cytokines whose overexpression causes lung injury. One of the anti‐inflammatory mechanisms of KKT would be reduction of the mRNA levels of toll‐like receptor 7 (TLR7) and myeloid differentiation primary response 88 (MyD88), which are essential for the production of inflammatory cytokines such as type‐1 interferons (IFN‐α and IFN‐β) and pro‐inflammatory cytokines such as IL‐6 and TNF‐α. Moreover, KKT treatment improved the Th1/Th2 imbalance which was mediated by the reduction of IFN‐γ and IL‐4. The Th1/Th2 imbalance is known to be related to the induction of inflammation [25].

Wu *et al*. revealed that KKT inhibited activation of the phosphatidylinositol‐3‐kinase (PI3K)/Akt signaling pathway in MDCK cells infected with influenza virus [26]. The PI3K/Akt signaling pathway is activated by viral infection including that of coronavirus, and supports viral replication [27, 28].

Kitamura reported that KKT treatment suppressed lipopolysaccharide (LPS)‐induced prostaglandin E_2_ (PGE_2_) production by decreasing cyclooxygenase (COX)‐1 activity, but had no effect on COX‐2. This activity was mediated by the suppression of extracellular signal‐regulated kinase (ERK) phosphorylation, which leads to production of cytoplasmic phospholipase A_2_ [29]. Additionally, another study reported the antipyretic activity in influenza‐infected mice through suppression of IL‐1α after administration of cinnamyl derivatives which are derived from *Cinnamomum cassia* bark, a component crude drug of KKT [30]. Therefore, KKT has potential anti‐inflammatory activity different from acetaminophen or non‐steroidal anti‐inflammatory drugs.

### Shosaikoto (SST)

SST, also known as Xiao‐Chai‐Hu‐Tang in Chinese and So‐Shi‐Ho‐Tang in Korean, is also widely used to treat the subacute phase of the common cold, pharyngeal tonsillitis, otitis media, bronchitis, gastritis, and hepatitis. The clinical indications of SST include chills and fever, anorexia, malaise, nausea, vomiting, cough, and dyspnea. SST extracts contain the following seven crude drugs: JP *Bupleurum* root, JP *Pinellia* tuber, JP Ginger, JP *Scutellaria* root, JP Jujube, JP Ginseng, and JP *Glycyrrhiza* [19]. SST is a typical formula used in the acute or subacute phase, which is called the ‘late yang stage pattern ^(^™^1)^’ [4].

SST has been reported to both decrease and increase IL‐6 production in lung injury. Ohtake *et al*. reported that oral SST administration decreased lung inflammation and increased lung IL‐6 in BALB/C mice treated with lipopolysaccharide (LPS)‐induced lung injury. Among the component ingredients of SST, Liquiritigenin contained in *Glycyrrhiza* led to increased production of IL‐6 *in vitro* [31]. Lipopolysaccharide is known to increase the activity of pro‐inflammatory cytokines such as IL‐6, TNF‐α, and IFN‐β. However, SST and its ingredients, oroxylin A and saikogenin D (components of *Scutellaria baicalensis* root and *Bupleurum falcatum* root, respectively,) decreased lung IL‐6 production in LPS‐treated C57BL/6 mice [32]. The authors noted that BALB/C and C57BL/6 mice had Th2‐ and Th1‐dominant immune systems, respectively. Th2 cells produce IL‐6, which is recognized as a pro‐inflammatory cytokine. These results suggest that SST may enhance IL‐6 production under Th2‐dominant conditions, whereas it decreases IL‐6 under Th1‐dominant conditions. Water extracts of *Scutellaria baicalensis* root also attenuated LPS‐induced lung injury and decreased NO production, TNF‐α, IL‐1β, and IL‐6 in LPS‐stimulated macrophages [33]. Another report revealed that SST and its ingredient wogonin‐7‐O‐glucronoside increased the CD4/CD8 ratio via a decrease in CD8(+) cells in anti‐CD3 antibody‐stimulated splenocytes. Wogonin suppressed CD8(+) T‐cell proliferation without inducing cell death [34]. Kang *et al*. reported that oral administration of SST enhanced the production of CD4(+) T‐cell cytokines including IFN‐γ and IL‐4 expression in the serum and spleen of anti‐CD3 antibody‐treated mice, which are an experimental T‐cell activation model. IFN‐γ activates the anti‐viral defense and IL‐4 enhances antibody production [35]. Therefore, SST may change the CD4/CD8 balance and modulate multiple cytokines against viral infection. Such multiple anti‐inflammatory and anti‐viral activities of SST may emerge from its constitution of multiple crude drugs.

In endotoxin‐induced inflammatory conditions, hyperactivated neutrophils induce acute liver injury through heme metabolism abnormalities. Sakaguchi *et al*. reported that SST administration improved hypoferremia and decreased δ‐aminolevulinate synthetase activity, heme oxygenase activity, and cytochrome p‐450 levels in the liver of LPS‐treated rats [36]. These results suggest that SST has a protective effect against acute liver injury.

SST and KKT have multiple reactive oxygen species (ROS)‐scavenging activities, especially against hydroxyl radicals and singlet oxygen [37]. Moreover, SST inhibits the production of NO from LPS‐treated murine macrophage cells in a dose‐dependent manner as mediated by NO scavenging activity [38]. Respiratory viral infections promote ROS production from epithelial cells and macrophages [39], with excessive oxidative stress of ROS being associated with acute lung injury [13]. The ROS scavenging activity of Kampo medicine may affect the pathogenesis of acute lung injury.

### *Platycodon grandiflorum* roots (PR, Kikyo)

Dried roots of *Platycodon grandiflorum* (Campanulaceae) are also known as kikyo in Japanese, jiegeng in Chinese, and doraji in Korean. In the original concept, kikyo is used for sore throat, cough, excessive phlegm, cough, and symptoms of respiratory inflammation. Some bioactive triterpenoid saponins in PR, including platycodin D, D3, and deapi‐platycodin, have been studied [40].

Shin *et al*. reported that platycodin D and D3 increased mucin release from rat and hamster tracheal surface epithelial cells *in vitro*, as well as from rat tracheas by nebulization [41]. Aqueous extracts of PR stimulated the secretion of mucin in a sulfur‐induced bronchitis model in rats. Platycodin D3 and deapi‐platycodin inhibited the production of MUC5AC mucin but stimulated the secretion of MUC5A mucin induced by phorbol 12‐myristate13‐acetate. These results suggest that platycodin D3 and deapi‐platycodin can inhibit *de novo* production of airway mucin but can stimulate the secretion of mucin produced under inflammatory conditions [42]. PR would have regulatory activities in airway mucin. Tao *et al*. reported that platycodin D decreased the total number of leukocytes, the percentage of neutrophils in lung tissue and BALF, the number of neutrophils infiltrating the pulmonary vessels, and alveolar lung injury induced by LPS and bleomycin while improving vascular permeability. Platycodin D inhibited the expression of inflammatory and apoptosis‐related proteins NF‐κB, caspase‐3, and Bax in the lung tissues, restored the expression of Bcl‐2, and improved superoxide dismutase activity. These results suggest that platycodin D inhibits acute lung injury and suppresses apoptosis [43].

Antiviral activities of PR have been reported in multiple phases of the viral life cycle. Zhang *et al*. reported that platycodin D suppressed type 2 porcine reproductive and respiratory syndrome viruses *in vitro* (PRRSV). Platycodin D inhibited PRRSV infection and reduced viral titers, non‐structural protein‐9 RNA levels, and N protein levels in a dose‐dependent manner in both the kidney cell line and primary porcine alveolar macrophages. PR inhibited PRRSV attachment cells, viral RNA replication, and viral release, as well as directly interacting with virions. PRRSV‐ and LPS‐induced cytokine (IFN‐α, IFN‐β, IL‐1α, IL‐6, IL‐8, and TNF‐α) production in macrophages was decreased by platycodin D [44].

PR has been shown to have immunomodulating and anti‐inflammatory activities. Polysaccharides isolated from the aqueous extracts of PR selectively increased the proliferation of B cells and activated iNOS transcription and NO production in macrophages, but did not stimulate T cells [45]. Choi *et al*. reported that aqueous extracts from PR stimulated macrophage proliferation and increased spreading ability, phagocytosis, cytostatic activity, and NO production in a dose‐dependent manner. PR stimulation increased levels of TNF‐α, IL‐1β, and IL‐6, as well as their mRNA transcription. PR also elicited dose‐dependent increases in NO and TNF‐α, which were due to an increase in inducible NO synthase and TNF‐α mRNA. Transient expression assays revealed that these activities are mediated by the NF‐κB transcription factor complex [46, 47]. On the other hand, in LPS‐activated RAW 264.7 macrophages, PR saponins, platycodin D, and D3 inhibited COX‐2, NO, and iNOS expression, but increased TNF‐α secretion, which was mediated by the reduction of NF‐κB and prevention of IκB expression [48, 49]. Aqueous extracts of PR also inhibited LPS‐induced NF‐κB nuclear translocation and expression of IκB in human airway epithelial cells [50]. Therefore, PR and its components would have immunostimulant and anti‐inflammatory activities.

### Gypsum (calcium sulfate, Sekko)

Gypsum is calcium sulfate dihydrate, which is also known as sekko in Japanese, shigao in Chinese, and seokgo in Korean. Gypsum is used for fever or heat (i.e., inflammation in the body), agitation, and thirst. In acute infectious diseases, gypsum is usually used together with *Ephedra* herb in decoctions. *Ephedra* herb alone has been found to increase rectal temperature, whereas the combination of gypsum and *Ephedra* herb reduced the rectal temperature in rats [51, 52]. The combination has antipyretic activity.

## DISCUSSION

In this review, we document the pharmacological activities of KKT, SST, PR, and gypsum, which are the components of SGT mainly for their use in viral infections or inflammatory conditions. These Kampo formulas and crude drugs have symptom‐reducing, antiviral, immunomodulating, anti‐inflammatory, and antioxidant activities (Table 2).

**Table 2:** Pharmacological activities of kakkonto, shosaikoto, *Platicodon glandiflorum* root, and gypsum

	Symptom‐reducing activities	Antiviral activities	Immunomodurating activities	Anti‐inflammatory and tissue‐protective activities	Anti‐oxidant activities
Kakkonto	•Increasing body temperature	•Reducing virus in the attachment, internalization and replication stage •Inhibiting PI3K/Akt signaling pathway (leading to inhibiting viral replication)	•Increasing phagocytic activity •Increasing IL‐12 in BALF •Decreasing IL‐1α, IL‐6, TNF‐α, IFN‐γ, IL‐4 expression •Decreasing TLR7, and MyD88 expression Improving the imbalance of Th1/Th2 cells	•Surpressing PGE2 mediated by decreasing COX‐1 activity •Improving influenza virus‐induced lung injury	•ROS scavenging activity
Shosaikoto	–	–	•Decreasing CD4/CD8 ratio (decreasing CD8+ cells) •Increasing/decreasing IL‐6 •Increasing IFN‐γ and IL‐4	•Decreasing lung inflammation induced by LPS •Protecting acute liver injury	•ROS scavenging activity •NO scavenging activity in macrophages
*Platycodon* root (kikyo)	•Increasing mucus secretion and diluting sputum •Enhancing the release of airway mucins	(Platycodin D) •Inhibiting infection, blocking attachment and internalization, •Inhibiting RNA replication, blocking progeny virus release, cell‐to‐cell spreading	•Activating B cells, increasing and activating macrphages •Increasing NO and TNF‐a in macrophages •Regulating the activity of NF‐κB (platycodin D) •Suppressing PGE2 production, inhibiting the induction of COX‐2 •Reducing cytokine gene expression (IFN‐a, IFN‐b, IL‐1a, IL‐6, IL‐8, and TNF‐a)	•Decreasing alveolar lung injury induced by LPS and bleomycin	–
Gypsum (sekko)	•Antipyretic activity (in combination with *Ephedra* herb)	–	–	–	–

**Figure 1:**
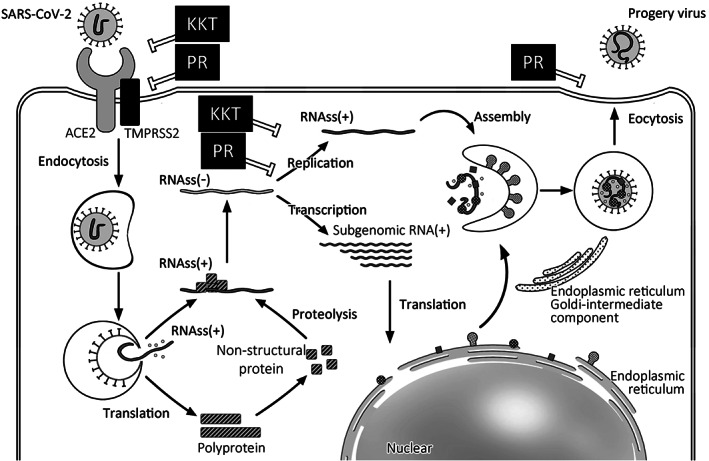
Life cycle of single‐strand RNA virus and possible activities of Kampo medicine.

It is important to evaluate if the Japanese original Kampo formula SGT used during the Spanish flu can also be effective against SARS‐CoV‐2. Coronaviruses, known for the crown‐like spikes on their surface (*corona* in Latin), are enveloped, single‐stranded positive‐sense RNA viruses belonging to the genus *Betacoronavirus*. Coronaviruses infect only mammals and cause respiratory symptoms similar to the common cold in humans and gastroenteritis in other animals [53, 54]. In the past two decades, two other highly pathogenic human coronaviruses have emerged — severe acute respiratory syndrome coronavirus (SARS‐CoV) and Middle East respiratory syndrome coronavirus (MERS‐CoV). SARS‐CoV has been isolated from bats, and MERS‐CoV has been isolated from camels. Genomic sequence research has shown that approximately 79% and 50% SARS‐CoV and MERS‐CoV sequences are shared with SARS‐CoV‐2, respectively, suggesting a similar mechanism of infecting human cells [55, 56]. The life cycle of SARS‐CoV‐2 is suggested to be similar to that of SARS‐CoV (Fig. 1) [56, 57, 58, 59, 60]. In brief, the spike proteins on the virus' surface attach to the receptor angiotensin‐converting enzyme 2 of the host cell and then employs the cellular transmembrane protease serine 2 for S protein priming. The virion subsequently invades the host cell through the endosomal pathway. The endosome is uncoated by endosomal acid proteases to activate viral fusion activity, and (+) strand genomic viral RNA is released into the cytoplasm. Two polyproteins are translated from the genomic RNA and are cleaved to generate 16 non‐structural proteins. Some proteins form a replicate–transcriptase complex, which acts as an RNA polymerase to synthesize (−) strand pre‐genomic RNA using genomic RNA as a template. Subgenomic RNAs are translated into structural proteins, including spike, envelope, membrane, and nucleocapsid glycoproteins, and are inserted into the endoplasmic reticulum and subsequently transported to the endoplasmic reticulum–Golgi intermediate compartment (ERGIC). Replicated genomic RNA and nucleocapsid proteins form nucleocapsids in the cytoplasm. The nucleocapsid coalesces with membrane‐bound components, forming progeny virions by budding into the ERGIC. A progeny virion is finally released from the infected cells through exocytosis.

Only a few studies have investigated the immune response to SARS‐CoV‐2 and the pathogenesis of COVID‐19. However, there are numerous studies on the previous highly pathogenic coronaviruses [60, 61, 62, 63], which are reported here. Invading viruses are recognized by the host innate immune system through pattern recognition receptors (PRRs), including Toll‐like receptors (TLRs) and RIG‐1‐like receptors (RLRs). TLR7 detects single‐stranded RNA, such as that of coronavirus and influenza virus on the cell surface or endosomal vesicles. RLRs are cytoplasmic receptors that detect viral genomic RNA. These recognition signals stimulate NF‐κB, mitogen‐activated protein kinase (MAPK), or interferon regulatory factor (IRF) pathways, which boost the production of type I interferon (IFN‐α and IFN‐β) and pro‐inflammatory cytokines such as interleukin (IL)‐6 or tumor necrosis factor (TNF)‐α [64, 65]. Autocrine or paracrine IFN‐α and IFN‐β signals activate the JAK/STAT signaling pathway and induce gene transcription, which exerts strong antiviral activities.

The adaptive immune response is an antigen‐specific response, which is generally initiated by antigen‐presenting cells (APCs), such as dendritic cells, macrophages, and B cells. APCs present small peptide antigens to naïve CD4(+) and CD8(+) T cells. Activated CD4(+) cells proliferate and differentiate into T‐helper (Th) 1, Th2, and Th17 cells. Th1 and Th17 cells promote the differentiation of CD8(+) T cells to cytotoxic T lymphocytes, which are capable of lysing infected cells. Th2 cells promote the differentiation of B cells. Activated B cells can act as APCs and can differentiate into plasma cells through the interaction of Th2 cells. Plasma cells secrete specific antibodies for the viral antigen, which further amplify the immune response [66].

Overreaction of the immune system, however, may lead to a cytokine storm and result in a severe manifestation of COVID‐19. Recent reports have shown leucopenia, lymphopenia, and reduction of both CD4(+) and CD 8(+) T cells in patients with COVID‐19 [67, 68, 69]. The number of T cells is negatively correlated with the levels of TNF‐α, IL‐6, and IL‐10. Although the triggers for the overexpression of inflammatory cytokines have not been elucidated, this phenomenon is correlated with the severity of COVID‐19. These immune responses cause several clinical symptoms in COVID‐19 cases, such as fever, cough, fatigue, myalgia, sputum production, shortness of breath, chest pain, headache, sore throat, dizziness, anorexia, diarrhea, nausea, and vomiting [10, 11].

According to the present review, KKT treatment increased body temperature, whereas the combination of gypsum and *Ephedra* herb decreased body temperature. High temperature reduced the replication of influenza A virus by affecting the function of acidic endosomes and inhibiting IL‐6‐mediated processes [70], which is mainly important during the acute phase of infection. On the other hand, a consistently high temperature can cause general malaise and fatigue. The amount of gypsum given is usually increased if patients have a high fever or strong inflammatory symptoms. Therefore, KKT may have antiviral effects in the acute phase, while gypsum may have antipyretic effects in the subacute inflammatory phase.

KKT and PR would have inhibitory effects against viral attachment, internalization, replication, progeny virion release, and cell‐to‐cell spreading with respect to influenza virus and HRSV (Fig. 1). In addition, glycyrrhizin, a component of *Glycyrrhiza* contained in KKT and SST, inhibits SARS‐CoV replication, absorption, and penetration *in vitro* [71].

**Figure 2:**
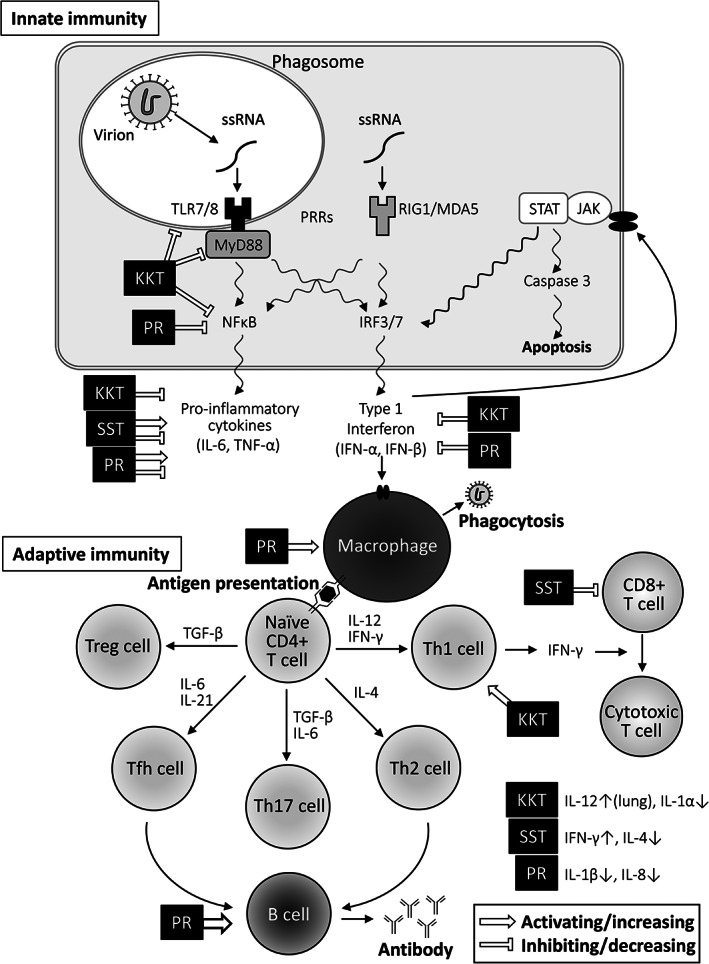
Immune response to viral infection and possible activities of Kampo medicine.

KKT, SST, and PR have multiple immunomodulating and anti‐inflammatory effects for protection against lung injury (Fig. 2). KKT has been shown to decrease IL‐6 activity. However, SST and PR were found to increase IL‐6 production even though KKT, SST, and PR could reduce LPS‐induced lung injury [25, 31, 32, 45, 46, 48]. IL‐6 is a pro‐inflammatory cytokine essential for viral infection, especially in its early phase [72, 73]. Protective effects of IL‐6 against coagulatory and hemostatic disturbances have been reported in LPS‐treated mice [74]. IL‐6 activates the JAK/STAT signaling pathway and enhances inflammation in viral infections, and also induces suppressor of cytokine signaling‐3 (SOCS3) which reduces JAK/STAT signaling. SARS‐CoV enhances IL‐6 production and decreases the expression levels of SOCS3 [75]. Moreover, the nucleocapsid protein of SARS‐CoV was reported to activate IL‐6 expression [76]. Subsequently, SARS‐CoV infection prolonged IL‐6 signaling, which would cause hyperinflammation and may even cause CRS. Appropriate regulation of IL‐6 may be needed in the different phases of viral infection and lung injury, with KKT, SST, and PR possibly playing different roles during different phases of infection. The response of T cells to Kampo medicine does not seem to be consistent. KKT increased IL‐12 and may increase the population of Th1 cells in the lung tissue, but does not change serum IL‐12 levels [24]. SST may decrease the population of CD8(+) T cells but do not change the number of CD4(+) cells in splenocytes [34]. The cytokine network regulates the differentiation and proliferation of different immune cells in different organs and in different phases of viral infections.

KKT, SST and PG have tissue‐protective effects against lung injury [25, 33, 43], and SST protects acute liver injury of LPS‐treated rats [36]. Histopathological examinations of lung biopsy from SARS [77, 78], MERS [79], and even COVID‐19 [80] patients revealed diffuse alveolar damage with reactive type II pneumocyte hyperplasia, along with intestinal fibrosis and chronic inflammatory infiltrates. Liver enzyme abnormalities are often observed in patients with highly pathogenic coronavirus infection, with ultrastructural and histological changes being similar to lesions found in viral infections [81]. Future studies are needed to investigate systemic and organ‐specific protective properties of Kampo medicine under inflammatory conditions.

In addition to having antiviral activity, the types of Kampo medicine investigated in this study have also been found to regulate the host immune system and to scavenge ROS, and may even allow for tissue protection. The multiple functions of Kampo medicine are due to the individual characteristics of its components.

Life cycles of single‐strand RNA viruses, which include influenza, HRSV, SARS‐CoV, MERS‐CoV, and even SARS‐CoV‐2, share a number of characteristics. Nevertheless, further studies are needed to investigate whether Kampo medicines may have similar antiviral activities on SARS‐CoV‐2. There have not yet been any reports on the use of KKT, SST, or PR for SARS‐CoV‐2. However, these medicines may be possible treatment options based on the homology among coronavirus, influenza, and RS viruses, which are all single‐stranded RNA viruses. The immune response to these viruses is also similar. Furthermore, recent *in silico* network analyses investigating the possible active components against SARS‐CoV‐2 showed that platycodin D in PR and liquiritin in *Glycyrrhiza* may be potential treatment agents [82, 83]. Further *in vitro* and *in vivo* studies are needed to investigate the activities of Kampo medicine against these viruses. A new method, like systems biology, would help to clarify the complex effects of Kampo medicine on the whole body.

Japanese reconstructive SGT did not contain PR (Table 1), but additional use with PR would be better for respiratory infectious diseases from both clinical and basic research perspectives. PR is typically used for the treatment of inflammatory diseases especially of the respiratory tract. Old Kampo doctors (e.g., Hakusho Kimura and Kyushin Yumoto) often use PR in addition to KKT or SST for severe inflammatory conditions [1, 84]. The results of this review support the therapeutic activities of PR against viral infection. Severe cases of COVID‐19 showed acute lung injury such as ARDS. Therefore, the combination of KKT, SST, PR, and gypsum, which compose SGT, may possibly be used as a treatment for COVID‐19.

Kampo medicine could be useful in terms of medical cost reduction. For example, maoto is effective for the treatment of influenza symptoms [85], and its medical cost for one patient (150 yen) is considerably lower than that using neuraminidase inhibitors (3260 yen) [86]. The current average prices of KKT, SST, and kikyosekko extract in Japan are 50, 156, and 50 yen per day, respectively (June 2020), and the price of these Kampo formulas has been kept low under the national health care insurance system. Cost–effectiveness analyses are to be expected in the medical practice of COVID‐19.

Kampo medicine should be used properly, with proper caution taken to prevent any adverse effects, although rare. For example, excessive and sustained use of *Ephedra* herb containing ephedrine alkaloids may induce palpitations, sleep disturbances, or urinary retention. Moreover, *Glycyrrhiza* may induce pseudoaldosteronism (hypokalemia, hypertension, and edema) [87, 88, 89]. In addition, SST may induce liver dysfunction and intestinal pneumonia in rare cases [90, 91]. Detailed information is provided in STORK. The conditions of the patients with respect to the adverse effects of Kampo medicine should therefore be carefully monitored.

The Chinese government proposed a treatment plan for COVID‐19, which involved an integrative approach combining Western and traditional medicine. Ren reported a clinically diagnosed case of COVID‐19 with pneumonia successfully treated with traditional Chinese medicine (TCM), and over 60,000 cases of COVID‐19 have been treated with TCM [92]. No clinical research has been reported on KKT or SST against viral respiratory infection, including coronavirus. It is necessary to conduct clinical trials investigating the effectiveness of Kampo medicine for COVID‐19 as well as basic research to investigate the activities of SARS‐CoV‐2. Even in the 21st century, we had been struck with other previous pandemics such as SARS‐CoV, MERS‐CoV, influenza A (H1N1)‐pdm09, and the recent SARS‐CoV‐2. Nevertheless, further research regarding the activities of Kampo medicines as well as countermeasures to prevent another viral pandemic, must be conducted.

## CONCLUSION

The combination of kakkonto, shosaikoto, *Platicodon grandiflorum* root, and gypsum, known as SGT, has potential therapeutic activities against single‐stranded RNA viruses, including SARS‐CoV‐2, based on viral homology. Further studies are needed to clarify the activities of Kampo medicine against SARS‐CoV‐2.

## CONFLICTS OF INTEREST

ST and TI belong to the Department of Kampo and Integrative medicine of the Tohoku University Graduate School of Medicine. The Department received a grant from Tsumura & Co., Japan. The grant was used according to the rules of Tohoku University. Potential conflicts of interest have been addressed by the Tohoku University Benefit Reciprocity Committee and managed appropriately.
